# Derivation, Functionalization of (S)-Goniothalamin from *Goniothalamus wightii* and Their Derivative Targets SARS-CoV-2 M^Pro^, S^Pro^, and RdRp: A Pharmacological Perspective

**DOI:** 10.3390/molecules27206962

**Published:** 2022-10-17

**Authors:** Vino Palani, Santhosh Chinnaraj, Murugesh Shanmugasundaram, Arunkumar Malaisamy, Viji Maluventhen, Vijaya Anand Arumugam, Kannan R. R. Rengasamy, Balamuralikrishnan Balasubramanian, Wen-Chao Liu, Maruthupandian Arumugam

**Affiliations:** 1 Department of Botany, Periyar University, Salem 636011, India; 2 Integrative Biology Division, International Centre for Genetic Engineering and Biotechnology (ICGEB), New Delhi 110067, India; 3 Department of Botany, Thiagarajar College, Madurai 625009, India; 4 Department of Human Genetics and Molecular Biology, Bharathiar University, Coimbatore 641046, India; 5 Laboratory of Natural Products and Medicinal Chemistry (LNPMC), Department of Pharmacology, Saveetha Dental College, Saveetha Institute of Medical and Technical Sciences (SIMATS), Chennai 600077, India; 6 Department of Food science and Biotechnology, College of Life Sciences, Sejong University, Seoul 05006, Korea; 7 Department of Animal Science, College of Coastal Agricultural Sciences, Guangdong Ocean University, Zhanjiang 524088, China

**Keywords:** (S)-Goniothalamin, characterization, COVID-19, M^pro^, spike glycoprotein, RdRp, molecular dynamics

## Abstract

The tracing of an alternative drug, Phytochemicals is a promising approach to the viral threats that have emerged over the past two years. Across the world, herbal medicine is a better solution against anti-viral diseases during pandemic periods. *Goniothalamus wightii* is an herbal plant, which has diverse bioactive compounds with anticancer, antioxidant, and anti-viral properties. The aim of the study was to isolate the compound by chromatography studies and functionalization by FT-IR, LC-MS, and NMR (C-NMR, H-NMR). As a result, the current work focuses on whether (S)-Goniathalamin and its analogue could act as natural anti-viral molecules for multiple target proteins viz., M^Pro^, RdRp, and S^Pro^, which are required for SARS-CoV-2 infection. Overall, 954 compounds were examined and the molecular-docking studies were performed on the maestro platform of Schrodinger software. Molecular-dynamics simulation studies were performed on two complex major compounds to confirm their affinity across 150 simulations. This research suggests that plant-based drugs have high levels of antiviral properties against coronavirus. However, more research is needed to verify its antiviral properties.

## 1. Introduction

The Coronavirus 2 or novel coronavirus 2019 disease related to severe acute respiratory syndrome (SARS) has been labeled a global pandemic, resulting in millions of deaths all over the world. Originating in China during the period of December 2019 [1], SARS- CoV virus is part of the family Coronoviridae and is an enveloped single-standard RNA virus [2,3]. Its genera are further subdivided into α, β, γ, and δ [4,5]. These viruses mainly affect the respiratory, gastrointestinal, and neurological functions of humans [6,7] and attack the alveolar epithelial cells, which results in the deactivation of the respiratory tract because the virus domain attaches to the receptors of the respiratory tract, known as angiotensin-converting enzyme 2 (ACE-2) [8,9]. The ACE-2 is highly expressed in humans, and it is a recognition site of SARS-CoV-2 [10,11]. The mechanism of the amplification of the microorganisms in humans can culminate in inflammatory responses and the release of a variety of molecules from macrophages, which are indispensable to fighting the disease [12,13]. Despite remarkable efforts, no vaccines or specialized therapies for COVID infection were initially developed. Other treatment techniques are the use of nucleoside analogues, remdesivir, and ritonavir, or anti-inflammatory agents that act on COVID-19 disease for emergency purposes [14,15]. Subsequently, researchers and scientists finally determined the best way to take herbal traditional medicine to overcome the disease. Moreover, the bioactive compounds derived from medicinal plants contain antiviral, antimicrobial, and anti-inflammatory properties. It is assumed that the immunomodulatory effects of these bioactive compounds are beneficial to treating or preventing COVID-19 disease [16]. *Goniothalamus wightii*, the Annonaceae family, is a small tree that is traditionally used by the kanikar tribes to treat rheumatism [17]. This species-based ligand acts as an anti-SARS-CoV-2 pharmacological agent, i.e., preventing the virus’ entry and increasing immune power.

The main structural and non-structural proteins present in the virus are the main protease (M^Pro^), spike glycoprotein (S^Pro^), and RdRp. These proteins were evaluated in large studies and showed interactions with the natural components which are derived from the medicinal herbal plants [18,19]. Furthermore, there has been a recent tendency in the computational world of large-natural-chemical-data retrieval to target various structures of coronavirus in order to define a good therapy. The complex pathophysiological mechanisms behind viral infections, as well as the associated side effects of currently available conventional treatments, need the establishment of a new drug-discovery process [15,20]. Finding a novel drug for the disease through conventional methods is extremely difficult and laborious. To overcome these drawbacks, the aim of the present study was to isolate and characterize the bioactive compound through chromatography analysis and functionalization by FT-IR, LC-MS, and NMR (C-NMR, H-NMR). Further, the compound extracted from *G. wightii* leaves against the anti-SARS-CoV-2-virus drug target was evaluated by molecular docking experiments conducted through an MD-simulation study.

## 2. Results

### 2.1. Purification of Hexane Extract of G. wightii

**Figure 1:**
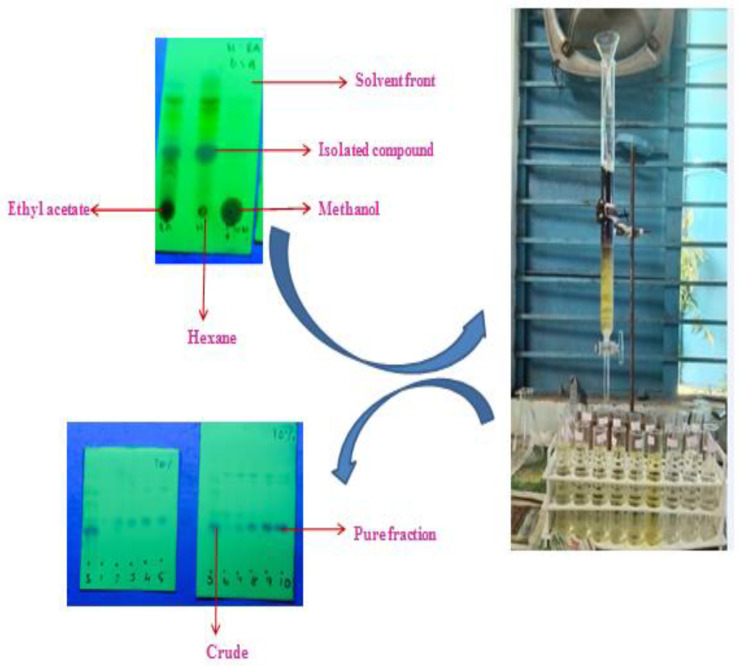
Separation of compound by column chromatography and analysis of TLC studies.

In this study, the TLC studies of the hexane–ethyl-acetate (6:4) of the hexane extract of *G. wightii* leaves revealed the presence of one major compound at Rf value 0.4. The compounds with Rf values of 0.01, 0.2, 0.7, and 0.8 were observed as a minor colored spot at the same solvent system. The column gradient eluted the expected product from the 10% hexane–ethyl-acetate system (Figure 1).

### 2.2. Characterization of Isolated Compound

**Figure 2:**
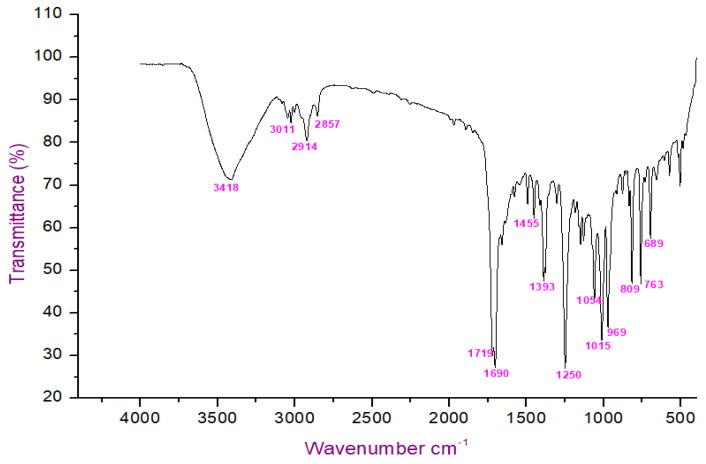
FTIR analysis of isolated compound.

**Figure 3:**
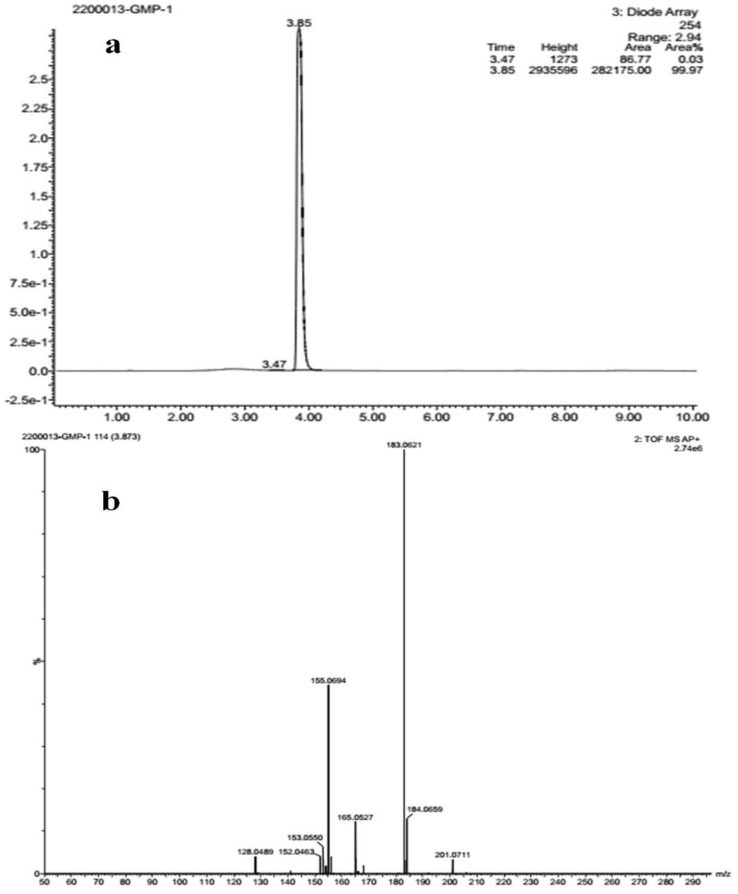
(**a**) Liquid chromatogram (**b**) mass spectrum studies of isolated compound.

The FT-IR analysis of the isolated compounds showed absorption bands and the wavenumbers (cm^−1^) of the prominent peaks at 3011 cm^−1^ were assigned to C-H aromatic stretching. The narrow and strong characteristic peaks at 2914 cm^−1^ belonged to CH2 asymmetry alkane (methylene) and the narrow and weak peak at 2857 cm^−1^ was assigned to CH2 symmetry alkane (methylene). The observed band at 1719 cm^−1^ was due to the C=O stretching frequency of the lactone ring. The peaks at 1690 cm^−1^ and 969 cm^−1^ were due to the C=C stretch and =C-H bend of the olefin group. The characteristic C-O stretching frequency of the lactone ring was observed at 1250 cm^−1^. The remaining peaks at 1455 cm^−1^, which were also weak, were assigned to the methylene CH2 bending of the alkane, and the 969 cm^−1^ mediums belonged to the C-H bending of the olefin. The presence of C=O, C-H, C=C, C-C, and C-O bonding structures were responsible for the presence of alkyl groups, aromatic groups, methylene groups, and olefin (Figure 2 and Table 1). The LC-ESI-TOF/MS-diode-array characterization of the compound by the ionization of the intensity time exhibited a single peak range at 3.85 and the compound purity was 99.97%. The spectrum of the proposed compound molecular formula, C_13_H_12_O_2_, was confirmed by LC-MS, which displayed a molecular weight of *m*/*z* 201 (M + 1) (Figure 3a,b).

### 2.3. Structural Analysis of Compound

The ^1^H NMR spectrum of the compound in the CDCl_3_ was recorded at 600-megahertz spectrophotometers and TMS (Tetramethylsilane) was used as an internal standard. The methylene hydrogens (–CH2–) of the lactone ring showed a chemical shift at δ 2.53–2.55 ppm as a multiple. The oxygen-attached proton (–CH–) of the lactone ring resonated at δ 5.09–5.10 ppm. The alkene protons showed chemical shift at δ 6.08 ppm (doublet of triplet), δ 6.90–6.93 ppm (Multiplet), δ 6.26 ppm (doublet of doublet), and δ 6.72 ppm (doublet). The aromatic-ring protons resonated at δ 7.26 ppm, δ 7.33 ppm, and δ 7.38 ppm. In the ^13^C-NMR spectrum of PD1, methylene carbon resonated at δ 29.9 ppm while oxygen-attached carbon was seen at δ 78.0 ppm. The signals observed downfield at δ 121.8, 125.7, 133.2, and 144.6 ppm were assigned to olefinic carbon.

**Figure 4:**
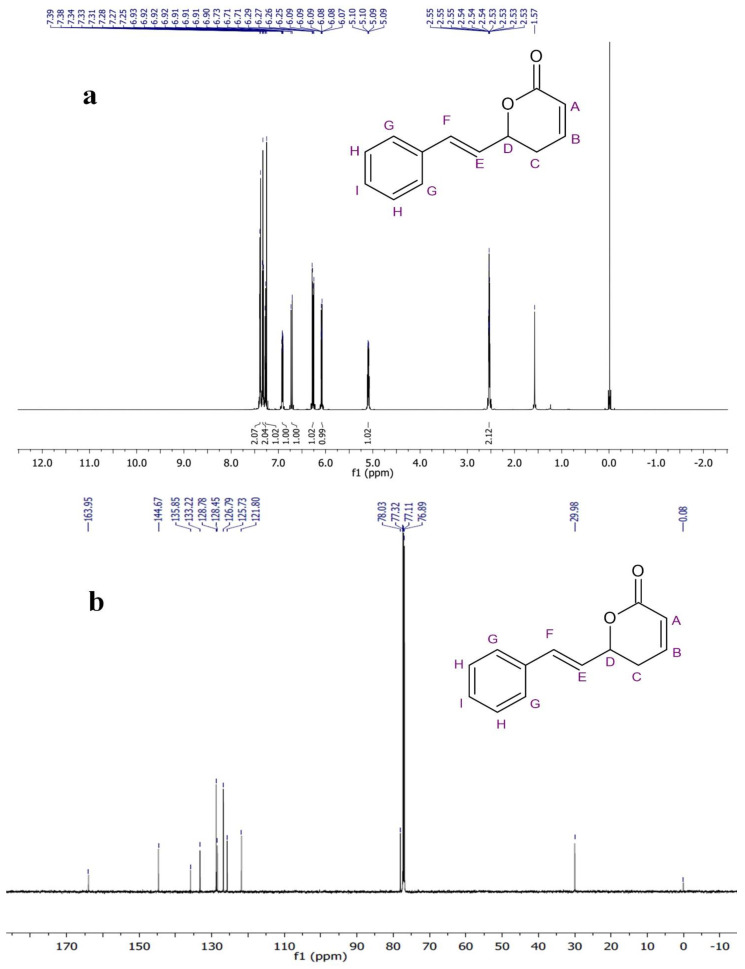
(**a**)^1^H-NMR and (**b**) ^13^C-NMR spectra of phytocompound.

The remaining aromatic carbons’ signals resonated at δ 126.7, 128.4, 128.7, and 135.8 ppm. The ^1^H and ^13^C-NMR data analysis showed the presence of carbonyl carbons, olefinic carbons, and a cyclic ring (lactone) (Figure 4a,b). The compound was identified as the (S)-Goniothalamin derivative, (E)-6-styryl-5,6-dihydro-2H-pyran-2-one. This is the first novel compound to have been isolated from *G. wightii* leaves.

### 2.4. Molecular Docking

**Figure 5:**
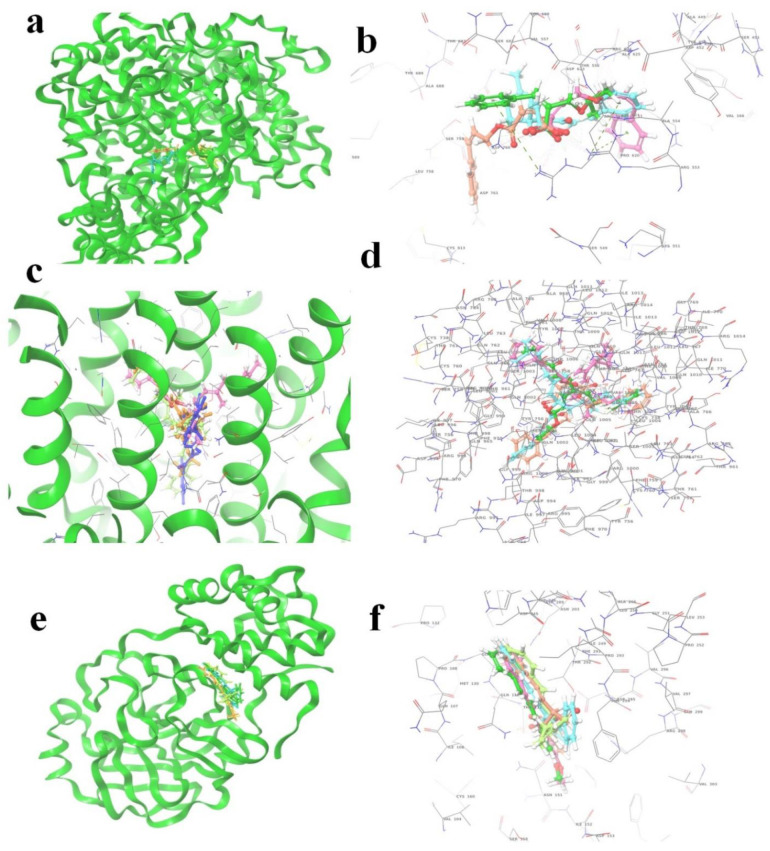
The 5 highest-ranked compounds shown in the docked position of target proteins, which occupies one pocket, and the three-dimensional structure is represented as (**a**,**b**) RDRP (R series), (**c**,**d**) spike glycoprotein (S series), and (**e**,**f**) main protease (M series). The ligand molecules are represented by the following colors: yellow (R1, S1, M1), orange (R2, S2, M2), sky blue (R3, S3, M3), magenta (R4, S4, M4), and purple (R5, S5, M5).

**Figure 6:**
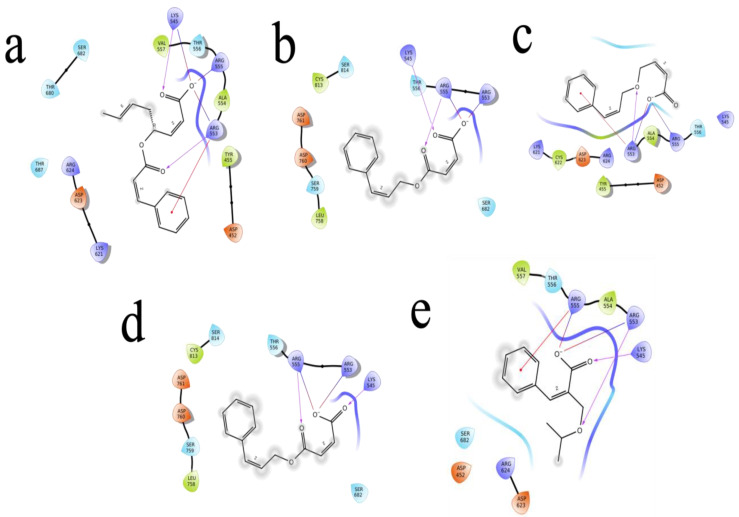
The 5 highest-ranked compounds represented with the RDRP protein of their two-dimensional interactive diagram as R1 (**a**), R2 (**b**), R3 (**c**), R4 (**d**), and R5 (**e**). Epoxy and carboxylic side chains exhibited the highest levels of interactivity among the top 5 structures.

**Figure 7:**
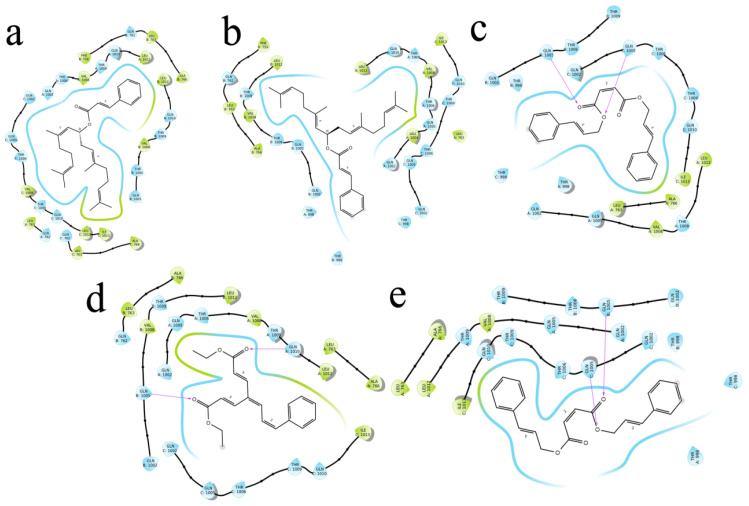
The 5 highest-ranked compounds with spike glycoprotein of their two-dimensional interactive diagram are represented as S1 (**a**), S2 (**b**), S3 (**c**), S4 (**d**), and S5 (**e**). Among the top 5 structures, epoxy-group side chains were highly interactive.

**Figure 8:**
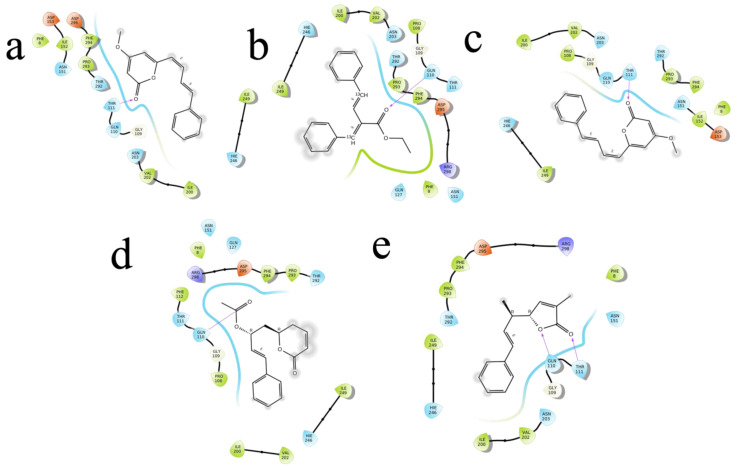
The 5 highest-ranked compounds with the main protease of their two-dimensional interactive diagram are represented as M1 (**a**), M2 (**b**), M3 (**c**), M4 (**d**), and M5 (**e**).

**Figure 9:**
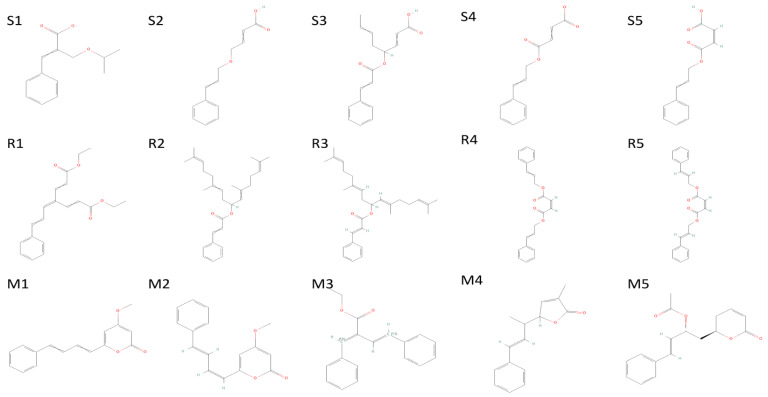
The 5 highest-ranked compounds of spike glycoprotein (**S1**–**S5**), RDRP (**R1**–**R5**), and main protease (**M1**–**M5**); their two-dimensional structures are depicted.

The (S)-Goniothalamin was docked with three enzymes, and showed the highest rank with the least binding score as −5.517 against spike glycoprotein, followed by −3.127 and −2.997 for the RDRP and the main protease, respectively. The compound against the spike protein showed amino-acid interactions of Leu763, Thr10069, and Thr1009 with bond-length distances of 2.45, 2.38, and 2.32, respectively. Next, the RDRP showed Asp623 (2.23, 2.28), Arg553 (2.58, 2.44), Tyr455 (2.44), Arg624 (2.41), and Thr 556 (2.07, 2.31, 1.78, 2.20, 2.22, and 2.38). Finally, for the main protease, Gln110, a distance of 2.36 was observed. The (S)-Goniothalamin with the analogous compound was docked with the multi-target proteins. Comparatively (S)-Goniothalamin showed a lower interaction-binding score than its derivatives. Here, it was found that the derivatives had more affinity than the compound. Further investigation of these compounds might be considered. Additionally, the top-ranked compounds were docked in the same pocket regions, which is how Figure 5 depicts their interaction. The two-dimensional diagrams in Figure 6 (RDRP), Figure 7 (spike glycoprotein), and Figure 8 (main protease) show how the molecule interacts with proteins. Finally, of the remainder of the five highest-ranked two-dimensional chemical structures of the compounds are represented in Figure 9.

### 2.5. Molecular-Dynamics Simulation

**Figure 10:**
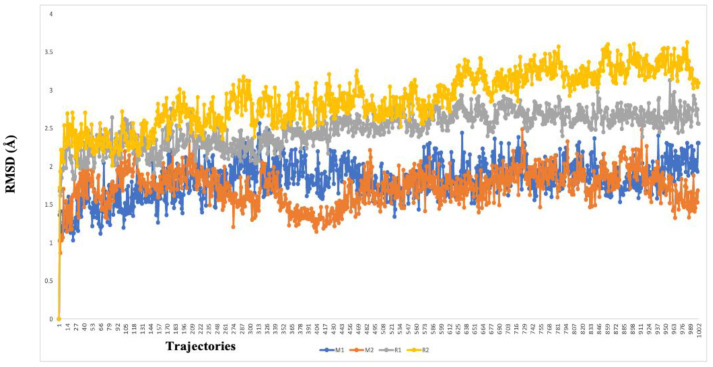
The RMSD graph of the two highest-ranked complex molecules of the main protease (M1—blue color, M2–orange color) and RDRP (R1—gray color, R2—yellow color). The deviation was below 3 Å in every instance, which suggests a higher affinity over the course of the 150-nanosecond simulation time.

**Figure 11:**
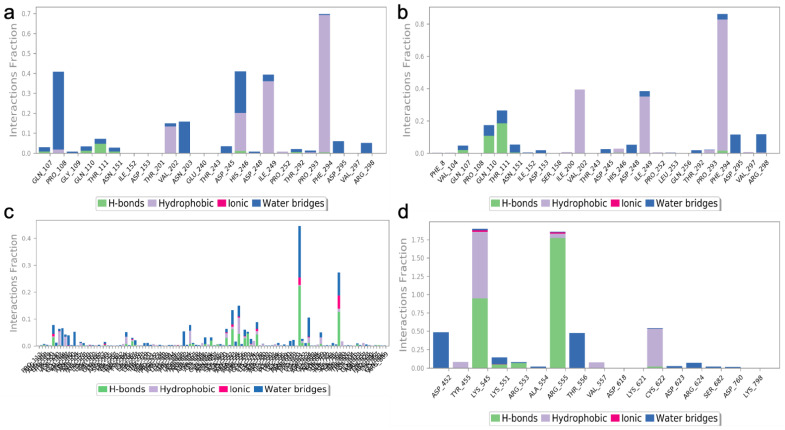
The protein–ligand contact of the two highest-ranked complex molecules of M^pro^ [M1 (**a**), M2 (**b**)] and RDRP [R1 (**c**), R2 (**d**)]. The bar graph with the appropriate colors indicates the type of interaction, and the interaction fraction represents the percentage of interactions. The normal baseline of interactions used to illustrate potential interactions is 0.3, which stands for 30% of the interaction.

The two highest-ranked docked-complex molecules were subjected for the MD studies to validate the affinity throughout the 150-nanosecond simulation time. In Figure 10, the RMSD of the complex molecules represented where the M1 and M2 denoted the two highest-ranked small molecules docked with M^pro^. The dynamics of both complexes were sustained at around 1.5 Å deviation, where the acceptable range is around 3 Å. The small R1 and R2 molecules showed a simulation around 2–2.5 Å, where they fell within the acceptable range. In the total simulation time, no major deviation was observed on the graph; by contrast, the simulation with the S^pro^ showed significant differences. The dynamics data were explained by the 1000 frame trajectories in the total simulation time. Table 2, showed that the main hydrogen-bond interactions showed the same patterns as those found in the MD simulation, where, in addition, the major hydrophobic interactions were found to have continuous contact. The contact represents the interaction. In the current study, the M1 and M2 complexed with the main protease showed hydrophobic interactions at PHE294, ILE249, and Val202. In the RDRP, additional hydrogen bonds and ionic and hydrophobic interactions were found at ARG555 and LYS545 in R2 and numerous amino acids showed interactions in R2 (Figure 11). With the evidence of the MD simulation data, the aforementioned compounds were shown to be promising candidates for further in vitro studies.

## 3. Discussion

Medicinal plants are attracting the attention of stakeholders all over the world. They are chemically diverse and can play an important role in the creation of novel drugs [14]. A plant contains numerous bioactive compounds with inhibitory potential for therapeutic applications [19]. Numerous diseases are caused by diverse harmful pathogens. Hence, we need a newly developed medicine against these diseases. The therapeutic remedy for human disease includes the purified compound of combined molecules from plant extracts and it is given to raise the range of antiviral ability against viruses, including infectious ones [21,22,23]. The isolation and purification of compounds based on TLC bioautography from crude extracts is used for biological assays. TLC-Bioautography has recently gained in popularity, owing to the fact that it is a simple, inexpensive, quick, and efficient technology that requires little laboratory equipment and apparatus and is compatible with a wide range of bioassays [24,25]. The present study’s TLC involved the separation of compounds with different solvent ratios. FTIR analysis is useful tool for the identification and characterization of compounds. It determines structures for various chemotherapeutic applications [26]. Currently, the FT-IR analysis of isolated compounds strongly supports the presence of lactone rings. The LC spectrum was used to analyze the bioactive molecules for isolation and discovery from plants [27]. The LCMS studies exhibited the particular compound in the 3.83 peak range for LC, confirming the proposed compound’s presence. The NMR spectrum of the present study’s ^1^H and ^13^C-NMR data analysis showed the presence of carbonyl carbons, olefinic carbons, and cyclic ring (lactone). Plant-extract compounds inhibit the multiplication of viruses in our body through RNA and protein synthesis, viral protease (3CL ^pro^, PL ^pro^), and the development of host-cell immunity, as well as by combating the viral lifecycle, viral attachment and penetration, and the mechanism of action of viral release [28,29,30]. Similarly, [31] reported the styrylpyrone derivative of goniothalamin from *G. umbrosus* through a cytotoxicity study of the antiviral activity of the DENV-2 (dengue virus) by time-removal assay. A reduction of 80% was observed at 24 h after pretreatment of styrylpyrone compound and their MD studied interactions with the E protein through hydrogen bonds and other bond interactions. The study by [32] reported the styryl lactone compound from goniothalamin found in *Goniothalmus* sp. A molecular-docking analysis found that protein binds the SCP-2 and enoyl-CoA hydratase, (3R)-hydroxyacyl-CoA domain of multifunctional enzyme type-2 through the formation of hydrogen bonds. The styrylpyrone is derived from styryllactone metabolites; these are isolated and purified only in the *Goniothalamus* sp. Annonaceae family. (S)-Goniothalamin is a styryllactone derivative, and it was applied to human-cancer-cell lines [33,34,35]. Goniothalamin is a natural substance that has been shown to trigger apoptosis in a variety of cancer-cell lines, including RT4 cells, HepG2 liver-cancer cells, and Chang cells goniothalamin, as well as acting against inflammatory diseases. Essentially, pre-treatment with NEM (N-ethylmaleimide) and NSAID (non-steroidal anti-inflammatory medicines) inhibits the inflammatory process, indicating the relevance of sulfhydryl substances and prostaglandins. Additionally, the study took the goniothalamin analogues to find a better drug candidate. Goniothalamin, a major component from the root and stem of *G. macrophyllus*, is a promising antitumor agent against the colon-cancer cell line, breast-cancer cell line, and large-cell lung carcinoma, despite showing more selective activity against the colon-cancer cell line and breast-cancer cell line than against large-cell lung carcinoma [36]. Furthermore, it has been shown to be a potential drug candidate for anticancer activities using cytotoxicity assay and antioxidant assay [37].

**Figure 12:**
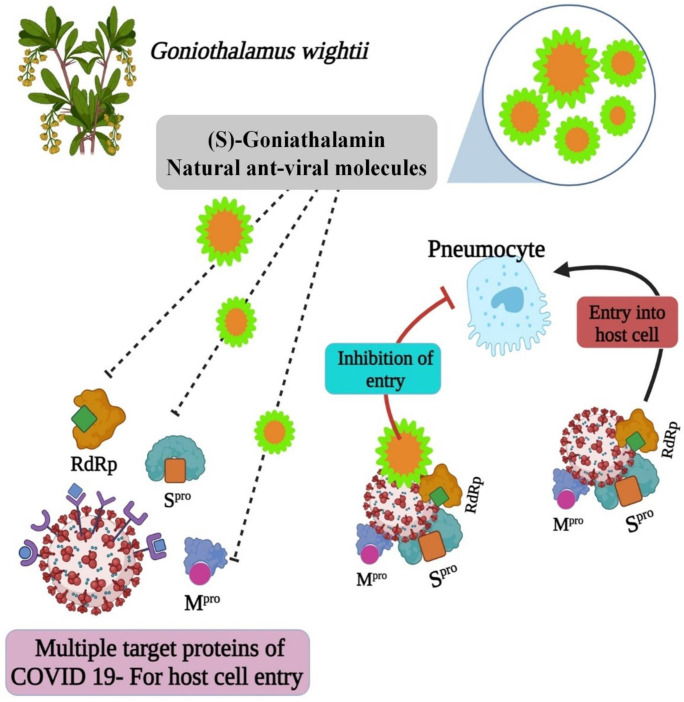
The mechanism of bioactive compound against SARS-CoV-2.

The molecule structures could provide insight into the development of anti-COVID medicines. Essentially, the best COVID-19 herbal medicine is made from natural ingredients alone or in combination. In this study, the purification of plant the biomolecule (E)-6-styryl-5,6-dihydro-2H-pyran-2-one, a derivative of (S)-Goniothalamin, is reported. The (S)-Goniothalamin interacted with selected proteins of the SARS-CoV-2 virus. The interaction-binding energy possessed negative variants in the range between −5.5, −3.127, and −2.917 Kcal/mol (Figure 5). The active binding scores suggested the potentially antiviral effect of (S)-Goniothalamin. This is the first finding of this compound’s activity against COVID-19. Therefore, the (S)-Goniothalamin compound was associated with these in silico docking results based on further expected effects of its pharmacological activities. According to [6], medicinal plants are sources of natural bioactive compounds against the COVID-19 viral disease. According to the present computational study, the purified molecule from plant extracts prevents the SARS-CoV-2 virus’ entry into the host (Figure 12).

## 4. Materials and Methods

### 4.1. Chemicals and Reagents

The chemicals, solvents, and reagents were of analytical reagent grade or the highest quality commercially available. They were purchased from Sigma, Aldrich, Avra, Fluka, and Spectrochem (Mumbai, India) and used as received without further purification.

### 4.2. Collection of Sample and Extraction

The *G. wightii* leaves were collected in Kalakad Mundanthurai Tiger Reserve Forest, Tirunelveli, Tamil Nadu, India. The plant was identified and verified at Ethnopharmacology and Algal Biotechnology Laboratory, Botany Department, Periyar University, India. To preserve the freshness of the plant leaves, a voucher specimen with the reference number PU/BOT/HVO.177 was submitted to the herbarium and immediately placed in an ice bag. Next, the plant-leaf material was subjected to shade drying for 15 days, after which the shade-dried plant material was subjected to pulverization to obtain fine powder, which was extracted in a Soxhlet apparatus using various solvents according to their polarity. About 50 g of leaf powder were extracted with 500 mL of hexane solvent. The hexane extract was filtered and dried by rotary evaporator. Finally, the crude was stored in desiccators until further use.

### 4.3. Isolation and Purification of Hexane Extract of G. wightii Leaves

The crude from n-hexane extract was purified by performing column chromatography. A dried glass column was used, and cotton plugs were placed at the bottom of the column. The column was loaded with silica gel (60–120 mesh). Next, the crude was taken in dichloromethane and it was mixed with silica gel for slurry. Subsequently, the resulting slurry was charged into the column. The column was eluted using a gradient–solvent system of hexane and ethyl acetate starting from 3% hexane–ethyl acetate. Fractions of 10 mL with each solvent system were collected and all the individual fractions were analyzed by TLC for homogeneity. The compound was collected as a pure fraction at 10% hexane–ethyl acetate. Next, the pure column fractions were subjected to the end product utilized for structure elucidation [38].

### 4.4. Characterization of G. wightii Extract

The end-product form of *G. wightii* was subjected to TLC using commercially available sheets. The sample was dissolved in ethyl acetate and then spotted onto the silica-gel plate and allowed to dry for a few minutes. Next, the plate was developed with n-hexane–ethyl acetate (7:3 and 6:4) and as a mobile phase in a previously saturated glass chamber. The developed TLC plate was air-dried under normal conditions and the spots were visualized under visible light and UV light (CAMAG REPROSTAR 3), as well as visualizing agent KMnO_4_. The 𝑅𝑓 (retention factor) values of identified compounds were calculated [39]. The isolated compound was characterized by using FT-IR (Perkin-Elmer spectrum). About 2 mg of the pure compound were mixed with KBr and well ground before preparing the pellet and the frequency range (ν) of 4000–400 cm^−1^ [40]. The liquid chromatography–mass spectrum (LC-MS, Waters-Synapt-G2) of the compound was analyzed by electrospray ionization (ESI) technique with 0.3 mL/min flow rate on the C-18 column run at 40 min. A total of 0.3 mg of compound was dissolved in 10 mL methanol and acetonitrile for the purposes of preparing the recording by mass spectrum [41].

### 4.5. Structural Analysis

The extract purified from *G. wightii* was measured in 600-megahertz NMR spectrometers at SASTRA University, Tanjore, where tetramethylsilane (TMS) is an internal standard. Next, 600 µL volume of the compound was transferred into a 5-millimeter NMR tube. The sample tube was inserted into the NMR and allowed to reach thermal equilibrium for 10 min before starting sample analysis. ^1^H and ^13^C nuclear magnetic frequencies were 500 and 125 MHz, respectively [42].

### 4.6. Computational Analysis

#### 4.6.1. Preprocessing of the Target Protein

The antagonistic effect of (S)-Goniothalamin derivatives on the functional protein of the SARS-CoV-2 virus was determined by molecular-docking studies. To this end, multi-targeted proteins were retrieved from the public database, Protein Data Bank, in a crystallographic structure, such as the main protease (PDB ID 6y2e), spike glycoprotein (6VYB), and RDRP (6M71). Using the protein-preparation wizard of the maestro, the preprocessing steps were carried out followed by assigning bond orders, adding hydrogens, creating zero-order bonds to metals and di-sulphate bonds, converting seleno-methionines to methionine, and filling missing side chains using. The structure was refined with optimization of hydrogen bond and minimization by applying the OPLS4 force field (Maestro, Schrödinger, LLC, New York, NY, USA).

#### 4.6.2. Ligand Preparation

The compound (S)-Goniothalamin extracted from *G. wightii* with its derivatives and analogous to a total of 954 compounds was retrieved from the PubChem databases in the 3D format of SDF (structure data file). The structure was refined using the LigPrep module in Schrodinger’s Maestro (v 12.8). The OPLS4 force field was applied, and 32 different states of stereoisomeric were derived (Schrödinger Release 2021-2: LigPrep, Schrödinger, LLC, New York, NY, USA, 2021). The processed ligand was used for further molecular-docking studies.

#### 4.6.3. Molecular Docking and MD Simulation

The target proteins and ligand molecules were docked using Maestro’s Glide docking module, with the flexible ligand-docking parameter enabled via Glide’s XP (extra precision) function. The docked ligand and protein interaction were examined using XP pose viewer to determine the optimistic pose, whereas the 2D-interaction diagram of a ligand–protein-complex molecule was obtained using the ligand-interaction module. Subsequently, the acquired XP pose was analyzed for the investigation of the binding interaction of ligand molecules upon the target protein [43]. From the 2 highest-ranked complex molecules, the MD simulation was performed using the Desmond module on the maestro platform of Schrodinger software. To create a hydration model, the protein-and-ligand complex was solvated in the 3D orthorhombic box with a buffer volume of 853,903 Å^3^ and a distance of 10 Å using a system-builder module (TIP3P water model). The MD simulation time was up to 150 ns utilizing the default Desmond settings and an NPT ensemble (constant number of atoms (N), constant pressure (P), and constant temperature (T). The simulation results were evaluated using a simulation-interaction-diagram tool, which supplied data on complicated macromolecule–ligand features of RMSD (root mean square deviation), protein –ligand contact, and timeline, to compute individual residue flexibility [4,44].

## 5. Conclusions

In this study, the (S)-Goniothalamin derivative was isolated and identified from *Goniothalamus wightii* leaves. The metabolites were further investigated through the (S)-Goniothalamin with their analogues docked with multiple proteins of SARS-CoV2; the lowest binding score was −5.517 against the spike glycoprotein, followed by −3.127 and −2.997 for the RdRp and the main protease, respectively. Incorporating biological sources to gather new and effective medication candidates could be a long-term strategy for enhancing the COVID-19-drug-discovery process. The molecular-docking results were further validated with MD simulation. The most frequently observed interactions in the simulation were hydrophobic, ionic, and hydrogen-bond interactions. These studies validated the methodology and the compound shown in the results. The methodology could therefore be considered as a drug-discovery approach with structural modifications. In this study, the (S)-Goniothalamin and its analogues surprisingly displayed more interactions in their similar structures, as shown by the 2D-interaction diagram. In future research, the efficacy of the reported compounds should be examined through in vitro studies.

## Figures and Tables

**Table 1:** FTIR spectrum of isolated compound using hexane extract of *G. wightii*.

Wave Number cm^−1^	Possible Functional Group	Standard Reference Range
3011	CH stretch (aromatic)	3000–3150
2914	CH stretch (asymmetric)	3000–2800
2857	CH stretch Aliphatic (symmetric)	3000–2800
1719	C=O stretch, lactone	1720–1750
1690	C=C stretch, olefin	1680–1640
1455	CH, bend, aliphatic	1465–1375
1393	C-C stretch, aromatic	1500–1400
1250	C-O stretch	1320–1000
1015	CH bend, Aliphatic (in plane)	1000–1250
969	=C-H bend, olefin	1000–650
763	C-H bend, Aromatic (out-of-plane)	900–675

**Table 2:** Molecular docking of (S)-Goniothalamin analogues, with multiple target proteins of RDRP (R), spike glycoprotein (S), and main protease (M) interaction, docking score, and their bond length are listed.

ID	Compound Name	PubChem ID	Glide Score	Glide Energy	Glide Emodel	Amino Acid Interaction	Bond Length
R1	3-Phenyl-2-(propan-2-yloxymethyl)prop-2-enoate	154129834	−6.874	−22.658	−25.405	Asp623, Arg624, Arg553, Thr556	2.38, (1.88, 2.41), 1.92, (2.36,2.43, 1.53)
R2	4-(3-Phenylprop-2-enoxy)but-2-enoic acid	76940553	−6.786	−26.902	−30.473	Arg624, Thr556	(2.43,1.67), 1.73
R3	4-(3-Phenylprop-2-enoyloxy)octa-2,6-dienoic acid	67934386	−6.679	−26.082	−31.825	Arg553, Thr556, Hie572	2.47,2.34,2.16
R4	4-Oxo-4-(3-phenylprop-2-enoxy)but-2-enoate	78478352	−6.569	−18.008	−19.319	Arg555, Thr556	(3.14,3.15), 2.74
R5	(Z)-4-oxo-4-(3-phenylprop-2-enoxy)but-2-enoic acid	70430994	−6.569	−18.008	−19.319	Arg555, Thr556, Lys545	3.73,(2.74,3.68),2.95
S1	Diethyl 4-(3-phenylprop-2-en-1-ylidene)hepta-2,5-dienedioate	71430013	−8.501	−47.499	−62.222	C-A Gln1005, Thr1009, Gln1002, Val1008 C-B Gln1002 C-C Thr1006	2.36 (2.16,2.76),2.44,(2.76,2.15),2.56,2.15 2.48
S2	2,6,11,15-Tetramethylhexadeca-2,6,10,14-tetraen-8-yl 3-phenylprop-2-enoate	54538910	−8.315	−33.738	−12.688	A Gln1005 B Gln1005, Thr1006, Gln1010,Thr1009, Gln762 C Gln1005	2.36 (2.16,2.76),2.44,(2.76,2.15),2.56,2.15 2.48
S3	[(6E,10E)-2,6,11,15-tetramethylhexadeca-2,6,10,14-tetraen-8-yl] (E)-3-phenylprop-2-enoate	67563707	−8.286	−44.473	−22.029	Gln1002, Thr1006, Gln1005, Val1008, Thr1009, Gln1005, Gln1002, Thr1009, Thr1006	(2.19,2.40),(2.26,2.23),2.36,(2.91,2.40),(2.36,2.35) (2.51,2.27,2.38),(2.34,2.31),(2.85,1.88) 2.25
S4	bis(3-phenylprop-2-enyl) (Z)-but-2-enedioate	141388166	−8.127	−45.087	−57.403	Thr1009, Leu763 Gln1002, Gln1005 Thr1009, Thr1006,Gln1002, Gln1010,Gln1005	2.39,2.57 2.37,1.87 (2.44,2.66),2.35,(2.01,2.35,2.59),(2.50,2.51),2.28
S5	bis[(E)-3-phenylprop-2-enyl] (Z)-but-2-enedioate	70543374	−8.127	−45.087	−57.403	Leu763, Thr1009 Gln1005 Gln1002, Gln1005,Thr1006, Thr1009, Gln1010	2.57,(5.29,2.39) 1.87 (2.37,2.33,2.01,2.59),2.28,2.35,(2.66,2.44),(2.50,2.51)
M1	4-methoxy-6-[(1Z,3E)-4-phenylbuta-1,3-dienyl]pyran-2-one	92528557	−4.755	−27.663	−36.773	Asn203, Gln110, Asn151	2.36,(2.31,1.91),2.34
M2	4-Methoxy-6-(4-phenylbuta-1,3-dienyl)pyran-2-one	322722	−4.723	−28.379	−33.967	Gln110, Asn151	2.16,2.30
M3	ethyl (E,2E)-4-phenyl-2-(phenyl(113C)methylidene)(413C)but-3-enoate	11482889	−4.399	−30.317	−39.043	Asn203	2.43
M4	3-Methyl-5-(1-methyl-3-phenyl-2-propenyl)furan-2(5H)-one	101575001	−4.382	−25.273	−28.44	Gln110	2.42
M5	(6R)-5,6-Dihydro-6alpha-[(2R)-2-acetoxy-4-phenyl-3-butenyl]-2H-pyran-2-one	100927498	−4.235	−28.978	−34.036	Thr111, Asp295, Gln110	(2.31,2.25). 2.28,1.76

## Data Availability

The data presented in this study are available on request from the corresponding authors.
